# Dosimetric Limitations in Treating Breast Cancer with Accelerated Partial Breast Irradiation Using Strut Adjusted Volume Implant (SAVI)

**DOI:** 10.7759/cureus.7528

**Published:** 2020-04-03

**Authors:** Ashraf Youssef, Anand Mahadevan, Adele Philippides, Heather Thieme, Angela Soto Hamlin

**Affiliations:** 1 Radiation Oncology, Geisinger/Holy Spirit, Mechanicsburg, USA; 2 Radiation Oncology, Geisinger Cancer Institute, Danville, USA; 3 Surgery, Geisinger/Holy Spirit, Mechanicsburg, USA

**Keywords:** savi, accelerated partial breast irradiation, breast cancer, dosimetry

## Abstract

We present one case of accelerated partial breast irradiation (APBI) using strut adjusted volume implant (SAVI) where there were limitations in delivering the dose as per the standard guidelines. The device was placed close to both the chest wall and the skin with little tissue surrounding the tip. Two plans were made in an attempt to achieve the standard therapeutic doses without over-treating the chest wall or the skin. Similar cases reported in the literature were reviewed. The dosimetry of the two plans was compared to the cases discussed in the literature.

## Introduction

Accelerated partial breast irradiation (APBI) with high dose rate (HDR) brachytherapy is one of the standard techniques for treating breast cancer [1-5]. One of the devices used is the strut adjusted volume implant (SAVI). It offers a unique ability of crafting the dose due to its multiple strut structure that can be loaded differentially [6-11].

## Case presentation

**Figure 1:**
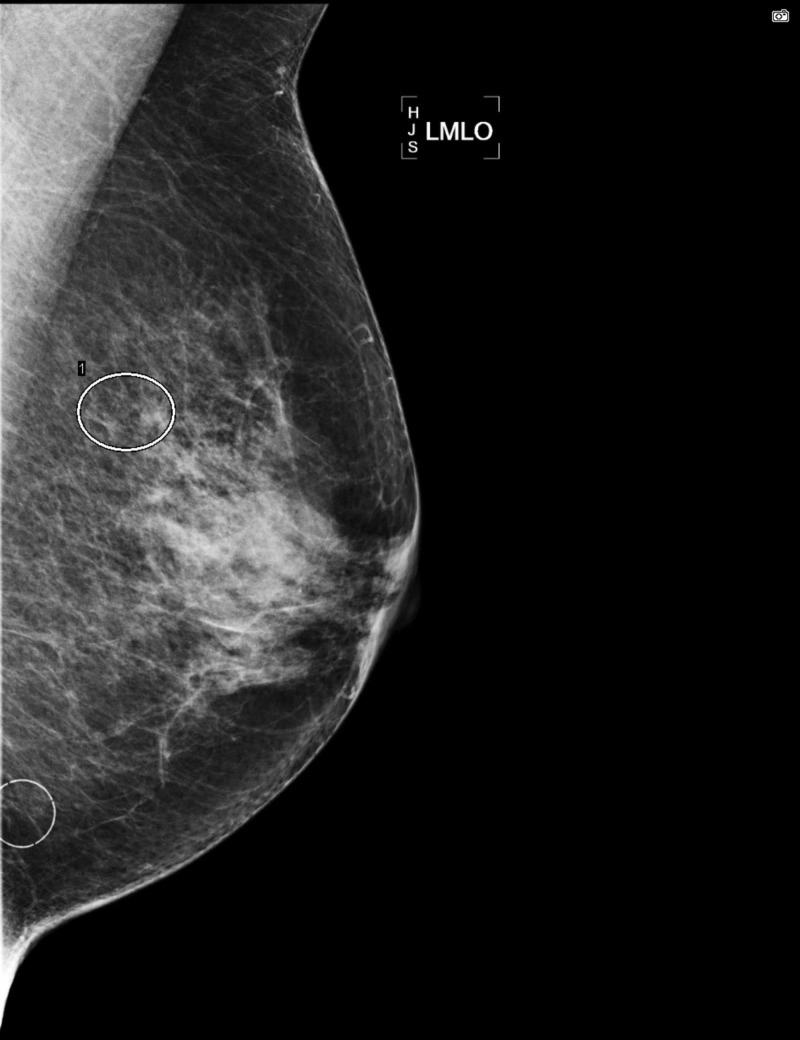
Mammogram of left breast, MLO view. MLO, mediolateral oblique

**Figure 2:**
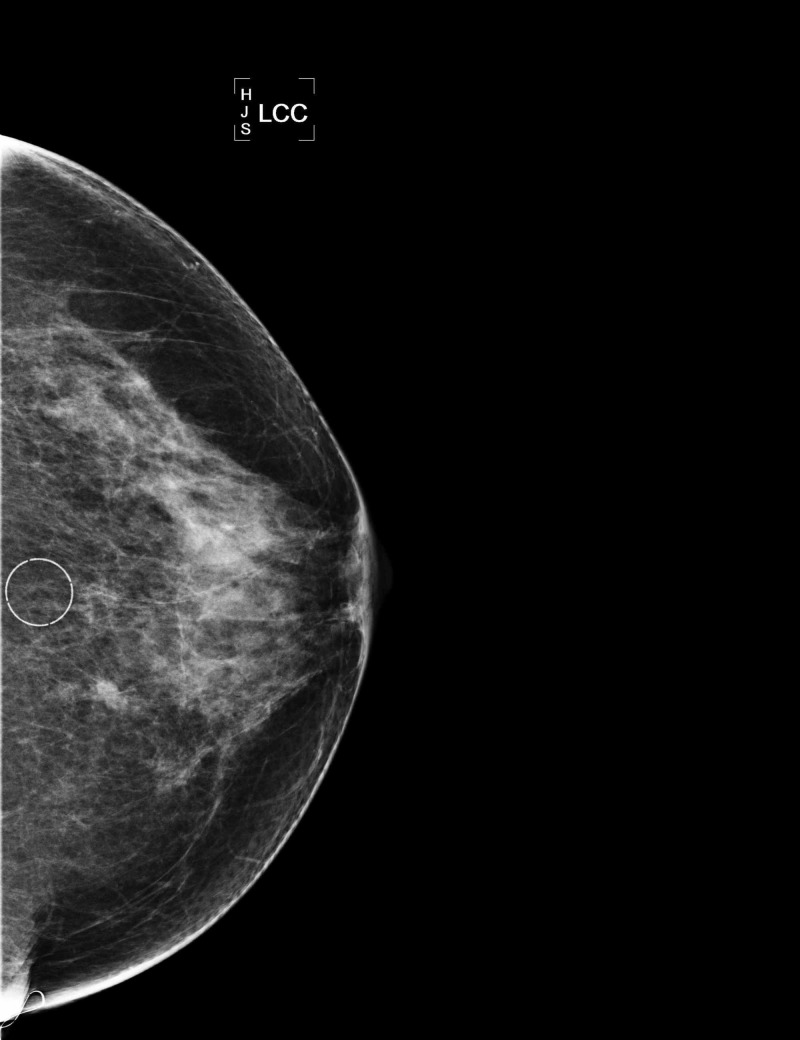
Mammogram of left breast, CC view with micro-calcifications circled. CC, cranio-caudal

**Figure 3:**
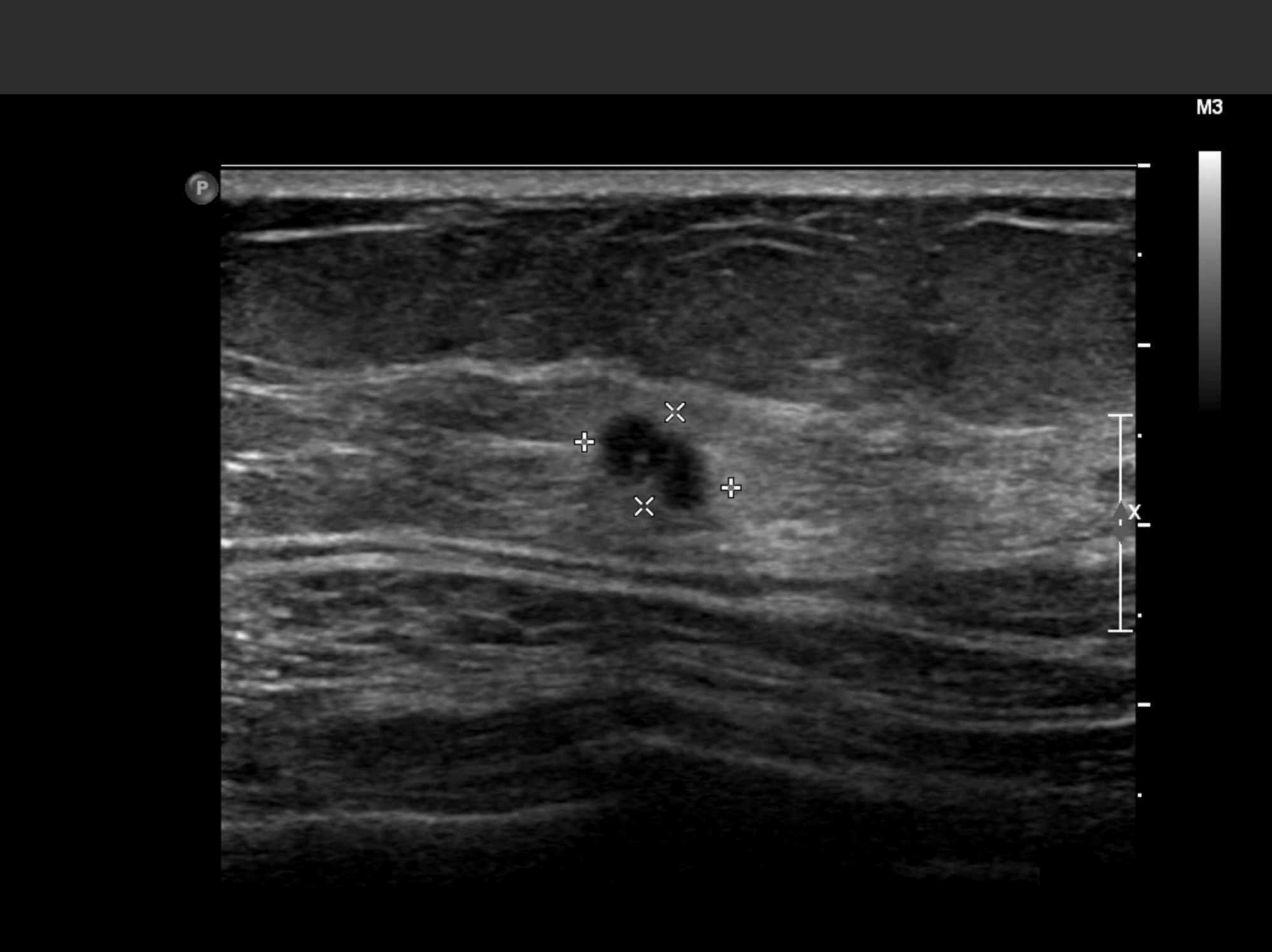
Left breast ultrasound image of the lesion.

Our patient was a 71-year-old female who had an abnormal mammogram. The mammogram showed a cluster of round calcifications in the upper inner quadrant of the left breast (Figures 1-3).

 An ultrasound showed a 6 mm x 6 mm x 4 mm hypoechoic; irregular shaped mass at 11 o’clock 4 cm from the nipple.

An ultrasound-guided vacuum-assisted biopsy showed invasive ductal carcinoma, grade 3 with ductal carcinoma in situ, high grade with comedo necrosis. The breast surgeon performed lumpectomy and sentinel lymph node biopsy. The final pathology showed 12 mm of invasive ductal carcinoma. All surgical margins were negative, and one sentinel lymph node biopsy was negative for metastatic carcinoma.

The SAVI Prep Catheter (SPC) was swapped by a “6-1 mini” SAVI device by the breast surgeon under ultrasound guidance. The patient had CT treatment planning study 48 h afterwards. The planning CT data set with 3-mm slice width was obtained with no gap between the slices. The CT images were sent to the Gamma med planning system.

The CT images were reviewed and exported to our HDR treatment planning system “Brachy-vision” and 3-D reconstruction was done. The cavity contour was drawn and 1-cm uniform positive expansion of “cavity” creates planning treatment volume (PTV). From the PTV contour, PTV_eval contour was drawn by subtracting the skin and chest wall (including the pectoralis muscle).

**Figure 4:**
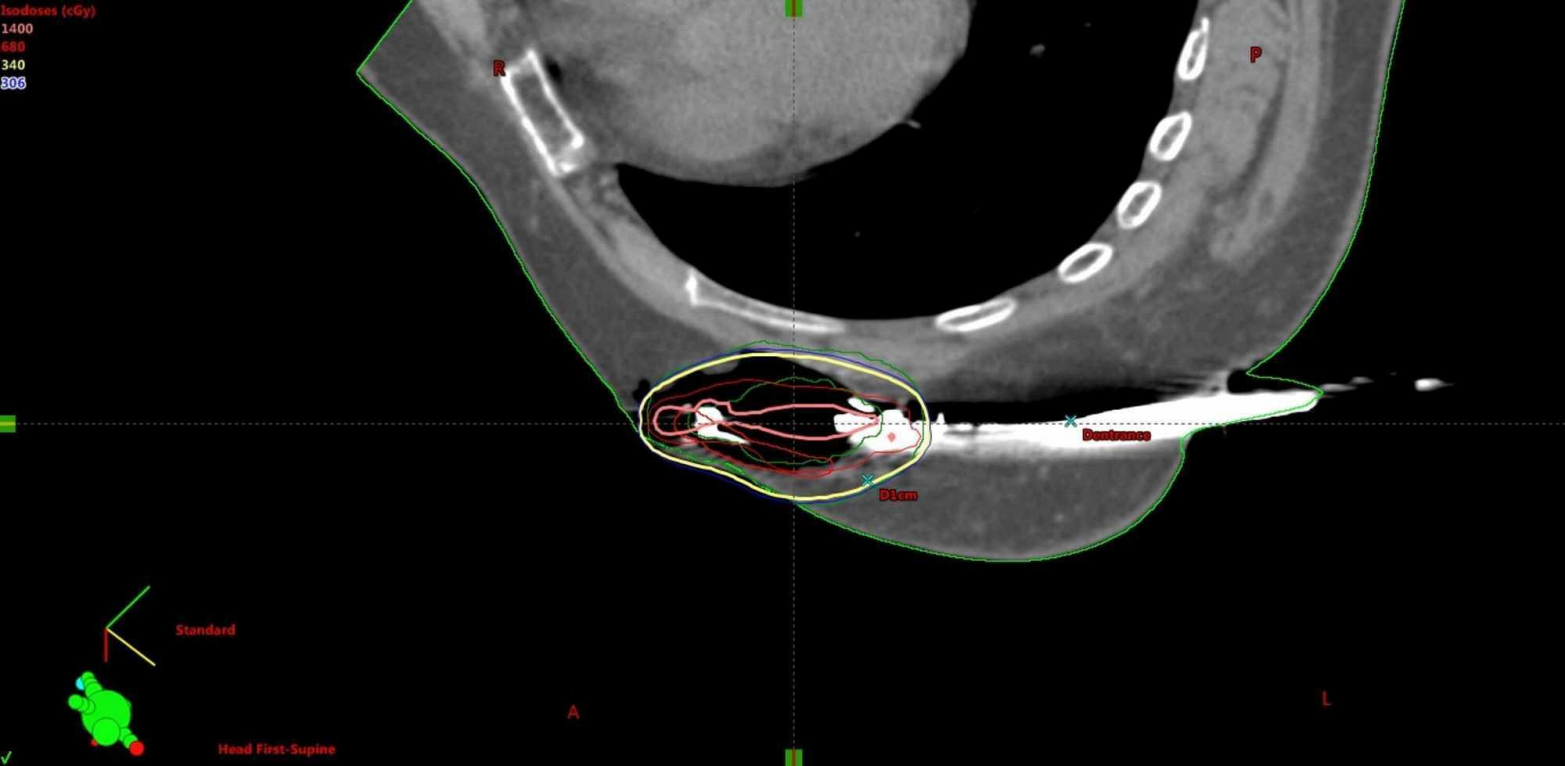
SAVI axial cut. SAVI, strut adjusted volume implant

**Figure 5:**
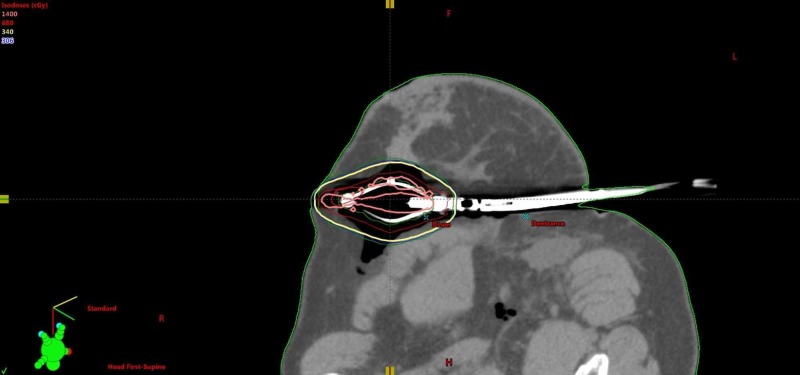
SAVI coronal cut. SAVI, strut adjusted volume implant

**Figure 6:**
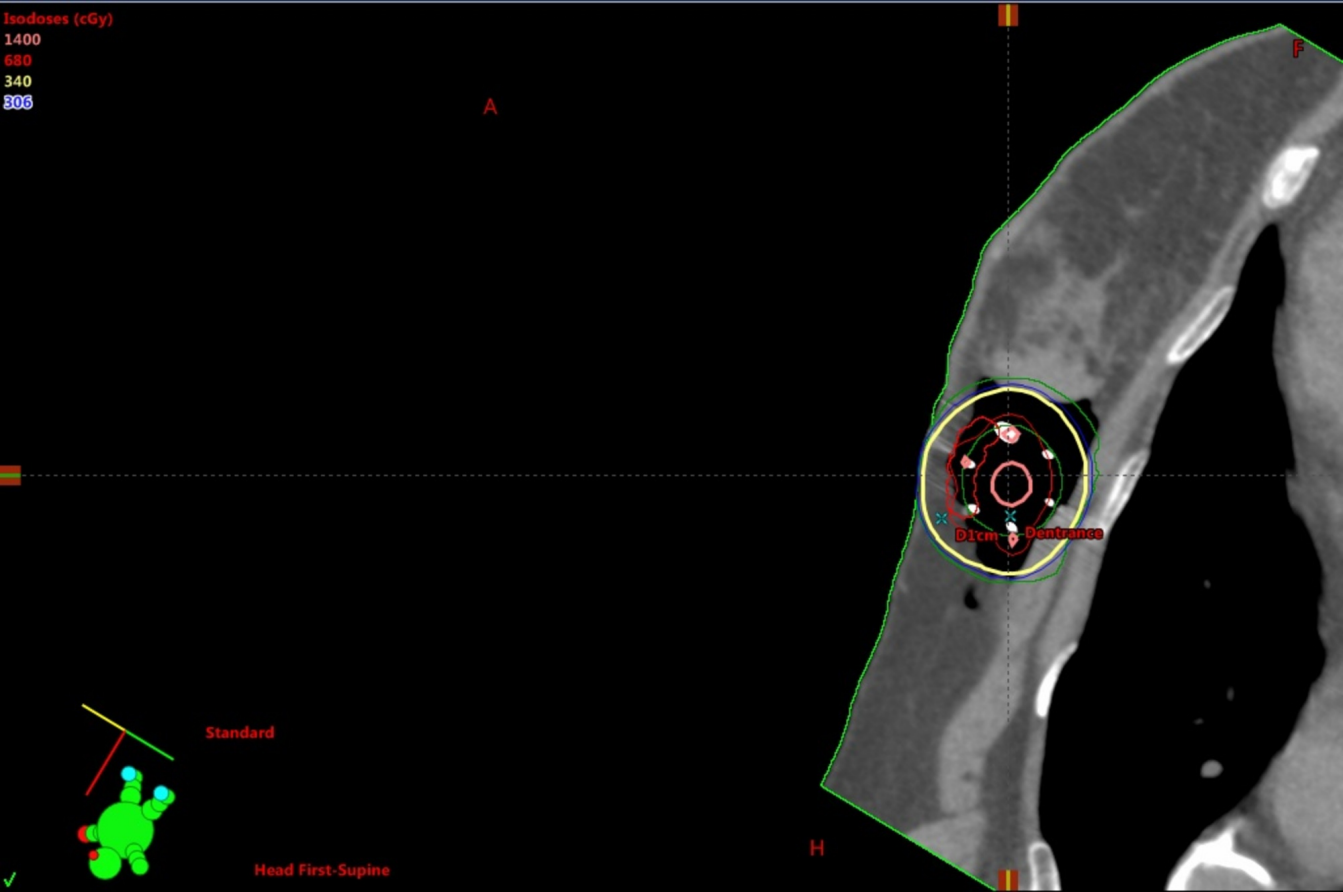
SAVI sagittal cut. SAVI, strut adjusted volume implant

A treatment plan was generated and optimized to achieve the following goals: 1) PTV_eval: 95% of volume receives ≥ 95% of the prescribed dose. 2) PTV_eval: ≥ 200% of dose must be < 20 cc absolute volume. 3) PTV_eval: ≥ 150% of dose must be < 50 cc absolute volume. 4) Skin dose should be < 100% of the prescribed dose. 5) Lung dose should be < 75% of the prescribed dose (Figures 4-6).

Many optimization attempts were made to achieve the treatment goals and two plans were made.

The first plan delivered 92.8% of the dose to 90% of the volume but the maximum skin dose was 2560 cGy (per fraction), and the chest wall dose was 529 cGy. The second plan delivered only 85% of the dose to 90% of the volume, but the skin dose was 647 cGy and the chest wall dose was 531 cGy. The two plans were unsatisfactory due to high doses to the skin and chest wall. As a result, a decision was made to remove the SAVI device (Table 1).

**Table 1:** Dosimetric values of plan 1 and plan 2. PTV, planning treatment volume

	PTV (cc)	90%	V100 (cc)	V150 (cc)	V200 (cc)	Max skin dose (cGy)	Chest wall (cGy)
Plan 1	44.25	92.8%	36	16	8	2560	529
Plan 2	44.25	85%	33.6	15	7.75	647	531

## Discussion

The SAVI device was designed to provide the radiation oncologist more room to carve the dose to match the patient anatomy. The multichannel property allows limiting the skin dose if the device is close to the skin. It also allows the prevention of overdosing the chest wall if it is placed close to the chest wall.

Manoharan reported a case where the cavity to skin distance was 4.5 mm and the SAVI was close to the pectoralis, a successful plan was able to limit the maximum skin dose to 190 cGy per fraction without compromising the coverage; the pectoralis dose was 425 cGy [8].

In a similar case reported by Sanderson, the skin to cavity distance was only 2 mm, and the cavity to rib distance was 4 mm. A successful plan delivered 96% of the dose to 90% of the volume and skin/chest wall doses were 346.6 cGy each [11].

 The difference in our case is that there was less tissue around the tip of the catheter as it was wedged between the skin and chest wall. We do not believe that a successful plan can be made if there is a) less than 5 mm around tip of the catheter, b) less than 5 mm separation between cavity to skin, and c) less than 5 mm separation between cavity and chest wall. A plan, however, can be made with 100% dose to the skin and chest wall but with less than optimal coverage to PTV_eval.

## Conclusions

The SAVI device has a unique design with multiple channels that allows a great adjustment of the dose in relation to the breast anatomy. We present a case where the dose coverage was compromised due to the anatomical position of the device between the skin and chest wall. We recommend that when the SAVI device is close to both the skin and the chest wall there should be more than 10 mm breast tissue at the SAVI’s tip.
